# High confidence copy number variants identified in Holstein dairy cattle from whole genome sequence and genotype array data

**DOI:** 10.1038/s41598-020-64680-3

**Published:** 2020-05-15

**Authors:** Adrien M. Butty, Tatiane C. S. Chud, Filippo Miglior, Flavio S. Schenkel, Arun Kommadath, Kirill Krivushin, Jason R. Grant, Irene M. Häfliger, Cord Drögemüller, Angela Cánovas, Paul Stothard, Christine F. Baes

**Affiliations:** 1 Centre for Genetic Improvement of Livestock, University of Guelph, Guelph, ON Canada; 2 Department of Agricultural, Food and Nutritional Science, University of Alberta, Edmonton, AB Canada; 3 Lacombe Research and Development Centre, Agriculture and Agri-Food Canada, Lacombe, AB Canada; 4 Institute of Genetics, Vetsuisse Faculty, University of Bern, Bern, BE Switzerland

**Keywords:** Genome informatics, Animal breeding, Genomics

## Abstract

Multiple methods to detect copy number variants (CNV) relying on different types of data have been developed and CNV have been shown to have an impact on phenotypes of numerous traits of economic importance in cattle, such as reproduction and immunity. Further improvements in CNV detection are still needed in regard to the trade-off between high-true and low-false positive variant identification rates. Instead of improving single CNV detection methods, variants can be identified *in silico* with high confidence when multiple methods and datasets are combined. Here, CNV were identified from whole-genome sequences (WGS) and genotype array (GEN) data on 96 Holstein animals. After CNV detection, two sets of high confidence CNV regions (CNVR) were created that contained variants found in both WGS and GEN data following an animal-based (n = 52) and a population-based (n = 36) pipeline. Furthermore, the change in false positive CNV identification rates using different GEN marker densities was evaluated. The population-based approach characterized CNVR, which were more often shared among animals (average 40% more samples per CNVR) and were more often linked to putative functions (48 vs 56% of CNVR) than CNV identified with the animal-based approach. Moreover, false positive identification rates up to 22% were estimated on GEN information. Further research using larger datasets should use a population-wide approach to identify high confidence CNVR.

## Introduction

Dairy cattle genetics has made great advances since the effects of single nucleotide polymorphisms (SNP) have been recognized on a wide range of mono or polygenic traits economically important for the dairy industry^1–5^. Genomic variation, however, is not only caused by SNP. Recent studies have shown that structural variants (SV) also have an important impact on phenotypes of a multitude of traits, such as milk production, reproduction, health, and feed efficiency^6–8^. Types of SV include translocations, inversions and copy number variation^9^. Copy number variants (CNV) form the most common class of SV in the human, plant and animal genome and can be identified as two types of event: copy number loss (CNL) or copy number gain (CNG). As the amount of DNA changes between samples with or without multiple copies of a segment, CNV are a type of the unbalanced structural variations^9^. Although the number of bovine CNV described in the literature is lower than the number of SNP, the fact that they have multiple alleles makes them highly informative^10^. The CNV can affect both monogenic traits, such as the coat color of cattle^11^, and polygenic traits such as feed efficiency, production traits, and reproduction traits of cattle^12,13^. For instance, a study by Liu *et al*.^14^ showed associations between CNV and production traits specifically in Holstein dairy cattle.

Identifying CNV is challenging and no consensus on the best method of identification has been reached because multiple factors, starting with the source of information on which the CNV are identified, influence the results. Moreover, most CNV identification methods were developed around the trade-off between high rates of discovery and low false positive rates. CNV have been identified in cattle from SNP genotyping arrays (GEN)^15–21^, hybridization arrays (ACGH)^14,22–25^, and whole-genome sequences (WGS)^26–31^. The degree of resolution of the different platforms leads to the identification of different CNV with varying lengths distributed unequally in the genome. Although the first studies defined CNV as genomic segments of 1 kilobase (Kb)^32^ or more, the latest developments in their identification on whole-genome sequences have reduced the minimum size of interest to 50 base pairs (bp)^33^. Generally, variants longer than 5 megabases (Mb) are considered erroneous and thus removed^13^. Variants detected with different methods, on different animals, and relying on different sources of information may overlap between 0 and 90 percent^12,26,34–37^. Moreover, Kommadath *et al*.^31^ suggested the method used for CNV identification has the most impact on the overlap between studies. The advantages and disadvantages of the detection methods on GEN and WGS data were reviewed by Winchester *et al*.^38^ and Pirooznia *et al*.^39^, respectively. One main limitation for all methods and sources of information is the quality of the reference genome on which the analysis is based. With a larger N50 contig size (26.3 vs 0.097 Mb) and a drastic reduction in the number of gaps (393 vs 72,051), the latest bovine reference genome assembly (ARS-UCD1.2)^40^ is a clear improvement compared to its predecessor UMD3.1^41^ and CNV are now expected to be possibly identified with more confidence. The marker density of the GEN data on which CNV identification relies also influences the set of CNV identified^42^. An estimate of the effect of the marker density on the false positive CNV identification rate, however, is lacking when relying on ARS-UCD1.2.

Methods based on WGS to identify CNV follow four different approaches: split read, read pair, assembly and read depth^9^. In contrast, the identification of CNV relying on GEN data is only based on the signal intensity and, in some cases, on the B allele frequency values generated at genotyping^38^. The signal intensity is a measure of the fluorescence intensity at the time of genotyping and reflects the quantity of DNA material present for a given probe on an array at the time of genotyping. The read depth approach in WGS detection approaches is its best equivalent. In both of these cases, the identification of CNV is indirectly based on the amount of DNA found for a region in a sample. CNV identification from GEN data has been developed to analyze the information of one individual at a time. To fairly compare CNV identified from these two sources of information, it is thus of importance to choose a single sample WGS identification method that relies on the read depth of the sequences. CNV regions identified based on two types of data and with two methods can be considered of high confidence^24^. The objectives of this study were: 1) to identify and describe CNV from GEN and WGS data based on ARS-UCD1.2; 2) to define sets of high confidence CNV regions following the two approaches and to find their possible impact(s) on traits of interest for the dairy industry through in silico functional analysis; 3) to compare the high confidence CNV regions with previously published variants; and 4) to estimate the effect of the marker density on false positive discovery rates.

## Material and Methods

### Animal material, genotyping and whole-genome sequencing

This study used only existing datasets and no animal samples were directly collected; all studies were conducted in accordance with the University of Guelph Animal Care Policy and Procedures, the provincial legislation and regulations of the Animals for Research Act, and the national guidelines and policies of the Canadian Council on Animal Care.

Both WGS and GEN data of 96 Holstein animals (15 cows and 81 bulls) were available. A total of 41 animals were sequenced within the Canadian Cattle Genome Project^43^ (CCGP), 32 within the Efficient Dairy Genome Project^31^ (EDGP), and 23 under the scope of multiple projects of the Vetsuisse Faculty of the University of Bern (CHE) and were primarily selected in order to identify causative variation for a wide range of diseases^44–46^. The Genetic Diversity Index method applied to select animals of the EDGP dataset for the sequencing was described in a simulation study^47^. DNA was extracted from frozen semen samples for the EDGP and the CCGP bulls. The WGS was performed using Illumina HiSeqTM 2000 (CCGP samples) or Illumina HiSeqTM X (EDGP samples). All analyses were carried out following the manufacturer’s protocols as described by Stothard *et al*.^43^. Paired-end reads were filtered, and the remaining reads were aligned to the latest bovine genome assembly (ARS-UCD1.2) using the Burrows-Wheeler Aligner^48^ (version 0.7.17) following the protocol of the 1,000 Bull Genomes Project (http://www.1000bullgenomes.com/, last accessed 2019-03-14). Reads bases were cut out with Trimmomatic^49^ (version 0.38.1) that were 1) identified as Illumina adapters, 2) at the start to the end of a read and had a quality lower than 20, and 3) not reaching a minimum average quality of 15 over a sliding window of 3 bp. After trimming, reads were dropped that did not reach an average quality of 20 and that were shorter than 35 bp. After alignment, duplicate reads were marked with the function *MarkDuplicates* of the Picard toolkit (version 2.18.15, https://broadinstitute.github.io/picard/, last accessed 2019-03-23) and base quality recalibration was performed with the functions *BaseRecalibrator* and *PrintReads* of the Genome Analysis Toolkit^50^ (version 3.8). The set of known variants provided by the consortium of the 1,000 Bull Genomes Project was used for base quality recalibration.

The same 96 animals that were sequenced as described above were also genotyped. Among them, 57 animals were genotyped with the Illumina BovineHD Beadchip (HD; 777,962 markers), 22 with the GeneSeek^®^ Genome Profiler Bovine 150 K (version 2 and 3; 150 K; 138,892 and 139,376 markers), 12 with the GeneSeek^®^ Genome Profiler Bovine HD (GGPHD; 76,883 markers) and 5 with the Illumina BovineSNP50 Beadchip (50 K; 54,001 markers). Thirteen additional Holstein bulls genotyped with the Illumina BovineHD Beadchip but not sequenced were included to analyze the influence of the genotype density on the CNV identification rates. The average gap sizes between the markers were 3.98Kb, 21.83Kb, 39.66Kb, and 66.75Kb for HD, 150 K, GGPHD and 50 K respectively. Markers with a GenCall (GC) score below 0.15 were removed on a per-sample basis. The SNP markers positions were updated to ARS-UCD1.2 using the map files available on the NAGRP data repository (https://www.animalgenome.org/repository/cattle/UMC_bovine_coordinates/, last accessed 2019-03-14).

### CNV identification and sets of high confidence CNV regions

The CNV were identified on all available WGS and GEN data on a per-animal basis. The CNV detection software PennCNV^42^ (version 1.0.3) was used to identify CNV from the GEN dataset (GEN_CNV). PennCNV relies on a hidden Markov model based not only on the Log R ratio (LRR) but also on the allelic ratio distribution (B allele frequency) of each sample. Prior to CNV identification and in order to reduce false positive results, the LRR values were corrected for genomic waves based on the Guanine-Cytosine content of the genomic regions 500Kb upstream and downstream of each investigated marker^51^. Only autosomes and markers with known position were considered for the analysis. After CNV detection, low-quality samples were removed from the analysis using the following cutoffs: LRR standard deviation above 0.3, B allele frequency drift above 0.01, and wave factor above 0.05. CNV covering less than 10 SNP in samples genotyped with the Illumina BovineHD Beadchip and less than three SNP in all other samples were also removed.

The CNV detection software program CNVnator^52^ (version 0.3.3) was used to identify CNV from the WGS data (WGS_CNV). This software partitions the read depth (RD) of the aligned genome for each individual over segments of a given length, corrects those depending on their Guanine-Cytosine content and then performs CNV identification. CNV detection was carried out by dividing the genome into segments of 200 bp. With this segment length, the ratio between the RD and its variance over all samples was 4.58, which fits the recommended ratio between 4 and 5 by Abyzov *et al*.^52^. After CNV detection, following recommended practice from previous studies^13^, variants shorter than 1Kb or longer than 5 Mb were removed. Only CNV from regions with a mean RD different from the RD average of the sample (P < 0.05, t-test) and with more than 50% of the reads mapped with a quality greater than zero were kept.

**Figure 1:**
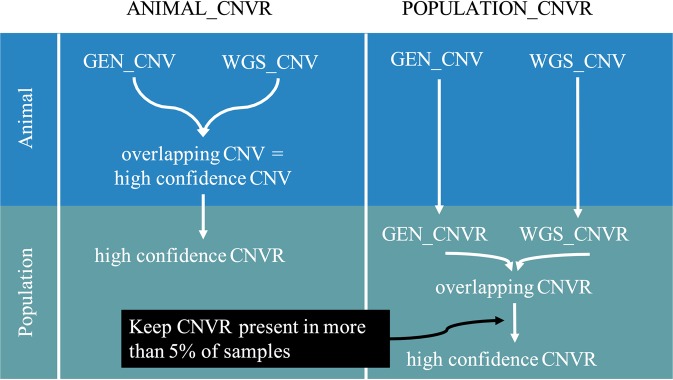
Steps to define high-confidence animal (ANIMAL_CNVR) and population (POPULATION_CNVR) copy number variant regions (CNVR).

Next, sets of high confidence CNV regions (CNVR) were created. The CNVR are formed by collating overlapping or contiguous CNV from GEN and WGS data. Merging CNV in regions was first described by Redon *et al*.^53^ and allows for population-wide CNV analysis. Two sets of high confidence CNVR were considered for analysis: ANIMAL_CNVR and POPULATION_CNVR (Fig. 1). To obtain the ANIMAL_CNVR set, CNV identified within the same animal from GEN and WGS data that had a reciprocal overlap of at least 50% of their length were considered high confidence CNV and merged to CNVR over all animals. All CNV identified with one data source that had any overlap were merged to create two sets of CNVR: the GEN_CNVR and the WGS_CNVR. GEN_CNVR and WGS_CNVR that reciprocally overlapped over at least 50% of their lengths and were present in more than 5% of the samples comprised then the POPULATION_CNVR set. CNVR that were found in some samples as CNG and in other samples as CNL were called MIX.

### Effect of the genotype array density on the identified CNV

The LRR and BAF from all 70 samples genotyped with the HD panel were masked down to the SNP overlap with the markers of the 150 K panel and of the 50 K panel. The three datasets were edited as previously described: markers with a GC score below 0.15 were removed, LRR values were corrected for the Guanine-Cytosine content of the 500Kb regions up- and downstream each investigated marker, and markers positioned on sex chromosomes or without known positions were removed. CNV were then identified using PennCNV^42^ and filtered as described earlier for each dataset. Finally, CNV remained, which were identified on all three densities for 30 samples composed the set for analysis. CNV identified on multiple SNP densities that had an overlap of at least one base pair were considered equal.

**Figure 2:**
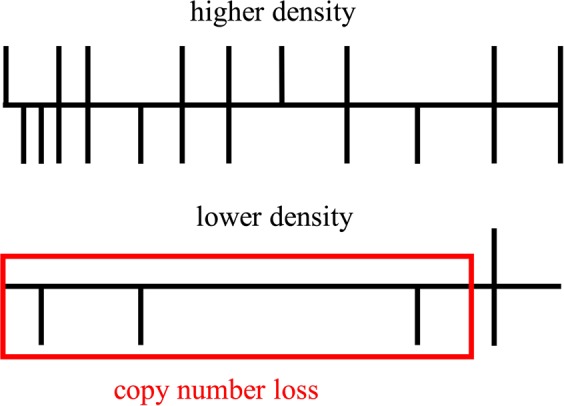
Copy number variants identified with a lower density marker panel but not with a higher density marker panel are considered false positive results. Vertical bars below the horizontal bar represent markers showing copy number loss. Vertical bars above the horizontal bar represent markers showing copy number gain. Vertical bars across the horizontal bar represents markers showing no copy number variation. In this example, three markers showing a copy number variation were needed to identify a variant.

GEN_CNV identification relied on the signal intensity value produced at genotyping; a higher signal intensity was equivalent to higher DNA concentration for a position, thus indicating a possible CNG or, in case the signal intensity was less strong than expected, a possible CNL. Filters were set at the time of CNV identification so that only regions where a minimum number of contiguous markers showing the same CNG or CNL pattern were considered CNV. Analysis of lower density marker panels could lead to the identification of CNV in regions where information of more markers in the same region would show no CNV pattern. CNV identified in those regions on lower density marker panel could thus be considered false positives. A situation where a CNL was identified with a lower density marker panel but not with a higher density marker panel is represented in Fig. 2. The upper part of the figure depicts a genomic region with a higher density marker panel, whereas the lower part depicts a genomic region with a lower density panel. In this example, a minimum of three markers had to show the same CNG or CNL pattern for the identification of a CNV. Vertical bars going through the genomic region represent markers with no CNG or CNL pattern, vertical bars only above or only below the genomic region represent CNG or CNL, respectively. The lack of marker information in the lower density panel across the genomic region led to the identification of a CNL that was not found in the higher density panel; such a CNL was considered a false positive hit in this study. False positive rates were computed per sample as the proportion of CNV found only with the 150 K or only with the 50 K over all CNV found with any density. Confidence intervals of 95% (95%-CI) for the false positive rates were computed with 10,000 times bootstrapping for the number of CNV identified and both false positive rates.

### Previously known variants

Two sets of previously known variants were considered for comparison with the high confidence CNVR identified in this study: the CNV deposited on the Genomic Variant archive database of EMBL-EBI (DGVa; https://www.ebi.ac.uk/dgva, last accessed 2019-03-24) and the CNVR identified on the datasets A, B, and C described by Kommadath *et al*.^31^.

The CNV identified and discussed in eight studies were available from the DGVa. Out of those, four studies identified CNV using GEN data^23,54–56^ and three studies identified CNV from WGS data^57–59^ and one study identified CNV from whole-exome sequence data^26^s. The number of samples of the studies varied from 6 to 539 and different breeds were included as well as *Bos indicus* animals (Table 1). Chromosome, start position, end position and type of all published CNV were retrieved and merged to form the DGVa CNVR dataset used for comparison with our results. Kommadath *et al*.^31^ identified CNVR in *Bos taurus* animals using the multi-sample approach implemented in cn.MOPS^60^ and relying on WGS data. They describe CNVR from four datasets with different numbers of samples of different breeds. As dataset D was the same as the 32 EDGP samples, only CNV identified on datasets A, B, and C of this study were considered. CNVR shorter than 5 Mb, and that were present in at least 5% of the samples of each dataset were retrieved and merged to form the CNVR set used for comparison with our results.

**Table 1:** Studies, data type, number of sample and number of breeds composing the CNV dataset from the Database of Genomic Variants archive.

Study by	Data type**	No. samples	No. breeds
Liu *et al*., 2010	GEN	90	17*
Hou *et al*., 2011	GEN	539	21*
Hou *et al*., 2012	GEN	472	1
Bickhart *et al*., 2012	WGS	6	4*
Boussaha *et al*., 2015	WGS	62	3
Keel *et al*., 2016	WES	175	20
Karimi *et al*., 2017	GEN	50	8*
Mesbah-Uddin *et al*., 2017	WGS	175	3

*Bos indicus animals were included along with Bos taurus in these studies.

**WGS – whole-genome sequence, WES – whole-exome sequence, GEN – genotyping array.

Both sets of known CNVR were generated based on the UMD3.1^41^ bovine reference genome but our results were based on ARS-UCD1.2. To allow for comparison between all sets of CNVR, coordinates of the previously described variants were translated to their ARS-UCD1.2 equivalent using the UCSC Genome Browser LiftOver tool^61^. Minimum ratio of bases that had to remap was set to 0.4, all other liftOver parameters were kept at their default values. In total and after translation to ARS-UCD1.2 positions, 9,169 CNVR composed the DGVa set and 4,525 CNVR were retrieved from the database of Kommadath *et al*.^31^. In all comparisons between CNVR sets, regions with a reciprocal overlap of at least 50% of their length were considered equal.

### In silico functional analysis

*Bos taurus* coding sequences located at the same genomic regions as the high confidence CNVR were retrieved from the Ensembl Genes database^62^ (ARS-UCD1.2, annotation release 96) with the Ensembl Biomart tool^63^. The OmicsBox (version 1.0.0; new updated software from Blast2GO^64^) was used to annotate the regions. The GO analysis was performed on the retrieved sequences taking into account the three GO categories separately (biological process, molecular function and cellular component) using OmicsBox^64^. Coding sequences were annotated with blastx and the OmicsBox mapping and GO annotation routines^65^. Nucleotide query sequences were compared against all the sequences found in the database of the National Center for Biotechnology Information (NCBI, https://www.ncbi.nlm.nih.gov, last accessed 2019-04-25). Matches between sequences of this study and the database were reported that reached a significance level of at least 0.001 (e-value) and had a similarity of at least 90%. No specific editing was made based on the sequence coverage. The GO significance levels were computed following Fisher’s exact test for multiple testing in OmicsBox. As described by Cánovas *et al*.^66^ and Li *et al*.^67^, the OmicsBox suite was also used to examine associated biological pathways involving the enzymes coded by the genes present in the high confidence CNVR based on the complete Kyoto Encyclopedia of Genes and Genomes^68^ (KEGG). GeneCards information was also retrieved for the identified and named genes^69^.

## Results

### Alignment and update to the bovine reference genome ARS-UCD1.2

The sequences of 96 Holstein animals were aligned to the bovine reference genome ARS-UCD1.2^40^. On average, 559,155,098 reads (standard deviation (SD) = 258,847,099) were obtained per animal, of which 96.6% (SD = 5.3%) were correctly mapped. Differences were observed depending on the sequencing project under which sequences were generated (Table 2). The average sequence coverage was of 20x and ranged from 8.57x to 42.53x. CCGP samples had an average sequence coverage of 12x (SD = 2x), EDGP samples had an average sequence coverage of 35x (SD = 4×), and CHE samples had an average sequence coverage of 15x(SD = 5x) [Suppl. Table 1].

**Table 2:** Summary of the whole-genome sequence dataset.

Sequencing project	Average no. of reads (SD*)	Average percentage of reads mapped (SD*)	Average read depth (SD*)	No. of samples
Canadian Cattle Genome Project	404,535,408 (70,157,276)	97.0 (1.1)	12×(2×)	42
Efficient Dairy Genome Project	903,140,899 (89,796,943)	97.6 (0.5)	35×(4×)	31
Vetsuisse Faculty	377,871,061 (118,817,596)	94.3 (10.5)	15×(5×)	23

*SD = standard deviation.

Positions of 85.4%, 86.2%, 90.4%, and 72.3% of the GEN markers on ARS-UCD1.2 were retrieved for the Illumina BovineHD Beadchip, the GeneSeek^®^ Genome Profiler Bovine 150 K, the GeneSeek^®^ Genome Profiler Bovine HD, and the Illumina BovineSNP50 Beadchip, respectively. Average percent of markers remaining after quality controls ranged between 68.5% and 88.5% (Table 3). The number of samples that passed the GEN quality filters limited the number of samples to 67 for both GEN and WGS datasets [Suppl. Table 2].

**Table 3:** Summary of the genotyping information dataset.

Genotype array	No. of markers	No. of remapped markers (% total)	Average no. of markers after QC* (% total)	No. of samples	No. of samples after QC*
Illumina BovineHD Beadchip	777,962	664,143 (85.4)	660,951 (85.0)	57	47
GeneSeek^®^ Genome Profiler Bovine 150 K	138,892	119,665 (86.2)	116,135 (83.6)	22	14
GeneSeek^®^ Genome Profiler Bovine HD	76,883	69,537 (90.4)	68,059 (88.5)	12	3
Illumina BovineSNP50 Beadchip	54,001	39,030 (72.3)	36,991 (68.5)	5	3

*QC = quality controls.

### Copy number variants identification

On average 7 GEN_CNV (min: 1, max: 70) and 1,855 WGS_CNV (min: 1,335, max: 4,413) were identified in 67 samples after quality control on a per sample and on a per CNV basis. The total number of CNV detected were 471 in GEN and in 124,302 in WGS. More CNL were found in both GEN and WGS data with 299 (63.4%) and 96,023 (77.2%) variants, respectively. CNV discovery in GEN was based on an average of 433,619 (SD = 280,740) markers. The data available for this study did not permit investigation of the effect of different read depths on the identified WGS_CNV. Average CNV lengths were of 132 Kb (min: 5 Kb, max: 1341 Kb) for the GEN set and of 22 Kb (min: 1.2 Kb, max: 4783 Kb) for the WGS set. The WGS_CNV were significantly longer than the GEN_CNV (P < 0.0001, Wilcoxon rank sum test with continuity correction). Once collated to CNV regions, the number of variants was reduced to 246 GEN_CNVR and 8,974 WGS_CNVR that had an average length of 111 Kb (min: 5 Kb, max: 1,787 Kb) and 54 Kb (min: 1.2 Kb, max: 13,296 Kb), respectively. The length distributions of the CNVR sets were not parametric and not different (P > 0.05, Wilcoxon rank sum test with continuity correction).

### Copy number variants and genotyping marker densities

**Figure 3:**
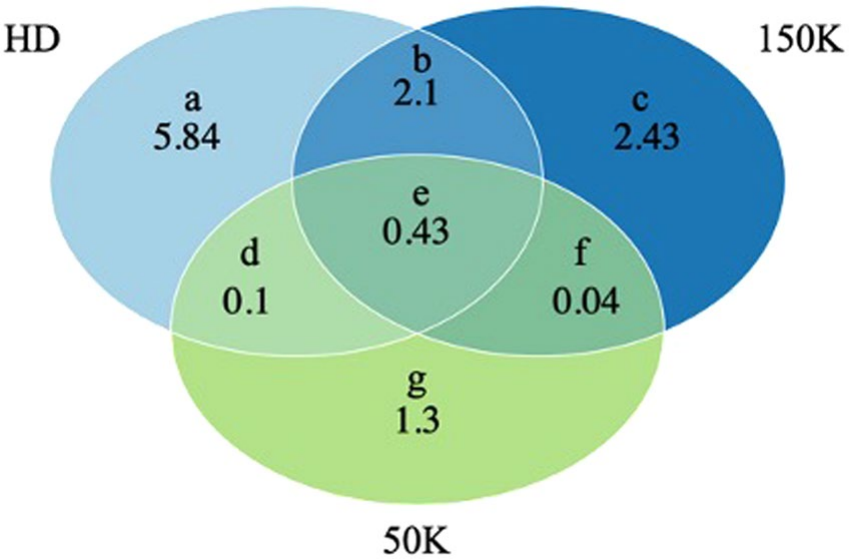
Average number of copy number variants (CNV) identified per animal based on the HD (777 K), 150 K, and 50 K marker densities. Letters represent the zones of the figures. False positive discovery rates were the ratio (c + f)/(all CNV) and (f + g)/(all CNV) for 150 K and 50 K, respectively.

Masking marker information of the animals with HD genotypes to 150 K and 50 K SNP panels enabled measurement of the effect of marker panel on the false positive CNV identification rate. Among the 70 animals with HD_GEN information, 30 passed all per sample-based filters and had CNV in each density. CNV lengths were similar to the CNV lengths reached with the complete GEN data, as previously described. On average, 8.451 CNV (95%-CI: 8.426, 8.476) were identified with the HD panel, 4.993 (95%-CI: 4.983, 5.002) with the 150 K panel, and 1.869 (95%-CI: 1.865,1.872) with the 50 K panel per animal. Average overlaps of CNV between set per sample are shown in Fig. 3. The CNV falsely identified with the 150 K marker panel composed on average 21.7% of the CNV identified with all densities (95%-CI: 21.6, 21.7) and 12.2% (95%-CI: 12.1, 12.2) of the CNV identified with 50 K marker panel were false positive.

### High-confidence copy number variant regions

**Figure 4:**
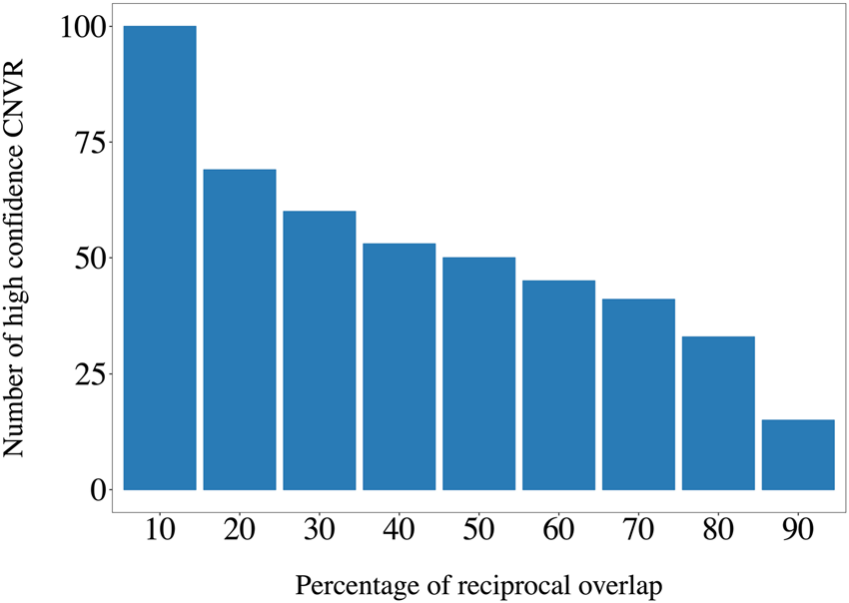
Number of high confidence copy number variant regions (CNVR) identified at the animal level when different minimum percentages were considered for the reciprocal overlap between the copy number variants identified using genotype array or whole-genome sequence information.

Altering the minimum percentage of reciprocal overlap between GEN_CNV and WGS_CNV to select high confidence CNV between 20 and 80 percent changed the number of variants considered to be of high confidence linearly. Low required overlap percentage (10%) or high overlap percentage (90%), however, had a larger impact on the number of high confidence variants, increasing (low overlap) or reducing (high overlap) it drastically (Fig. 4).

**Figure 5:**
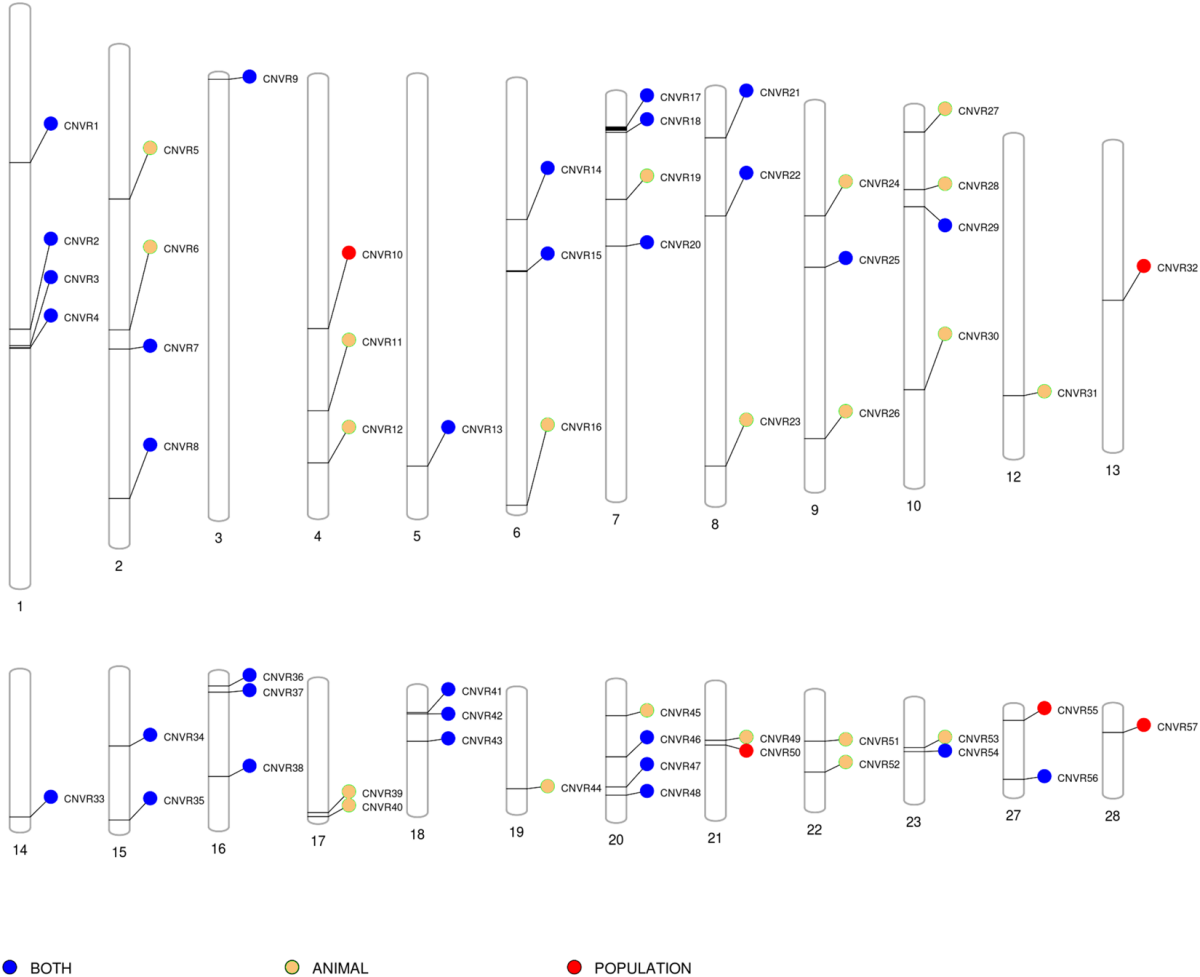
Distribution of the high confidence copy number variant regions (CNVR) across the bovine genome found in different sets of CNVR. Only chromosomes carrying an identified variant are pictured.

**Figure 6:**
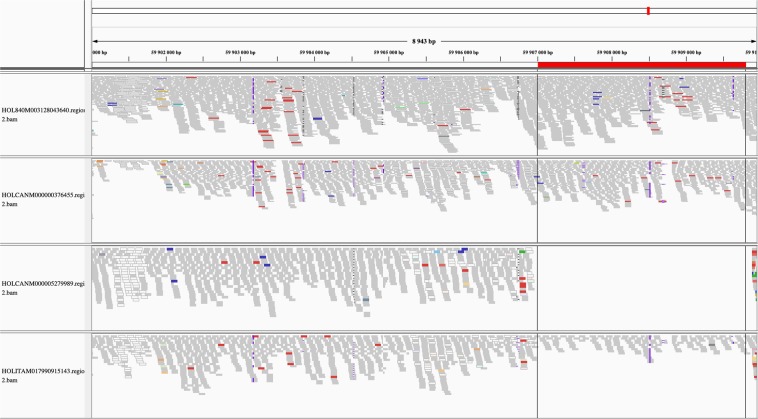
Read alignments of four samples on *Bos taurus* autosome 20 from 59,901,001 base pairs (bp) to 59,910,000 bp belonging to the unique high confidence copy number variant (CNV) region no. 48. The two top samples have no predicted CNV in the region under the red bar, whereas the two bottom samples have a copy number loss predicted.

**Figure 7:**
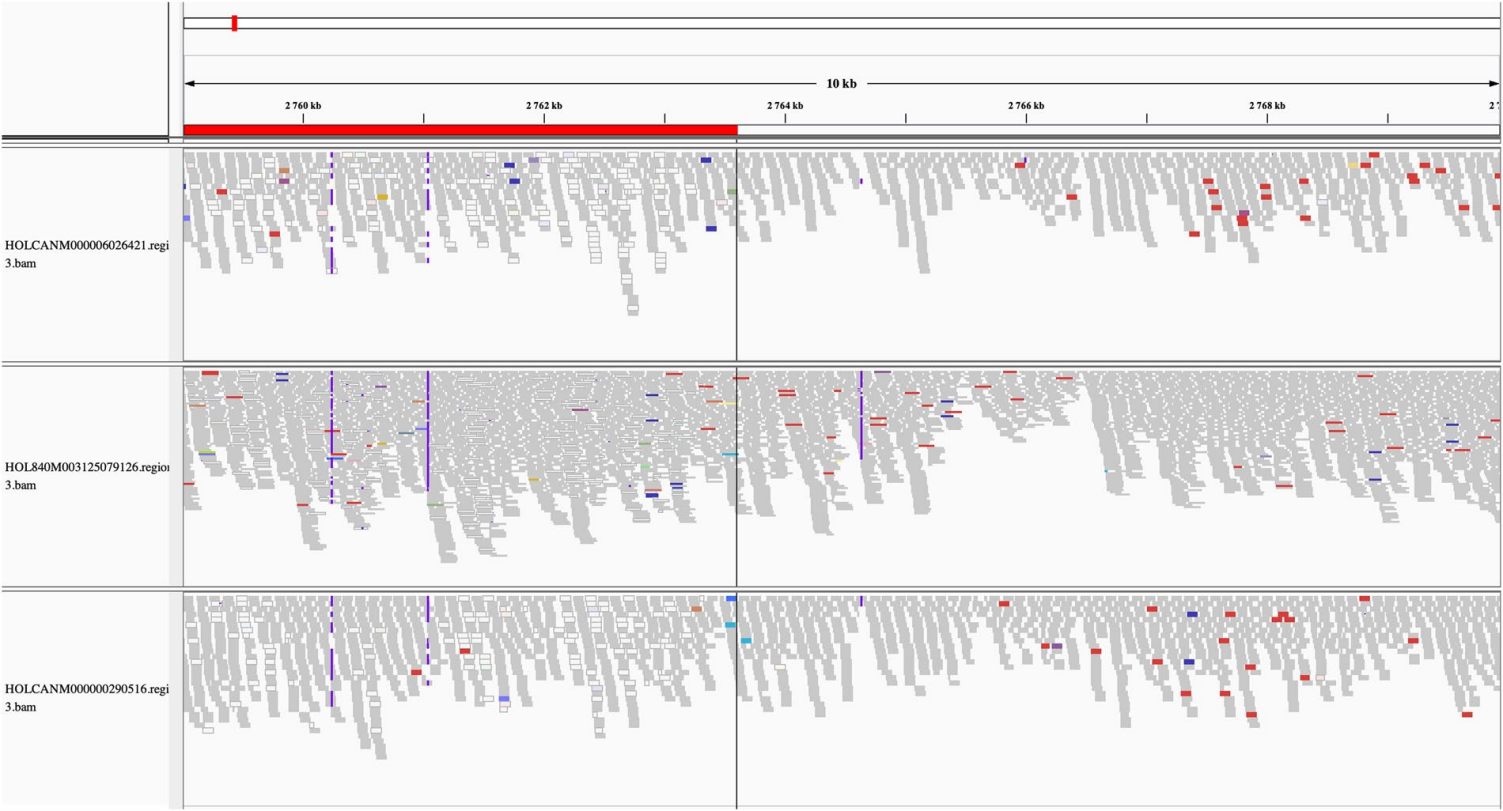
Read alignment of three samples on *Bos taurus* autosome 20 from 2,759,000 base pairs (bp) to 2,770,000 bp. Although a copy number gain is predicted for the top sample for the region under the red bar, no difference in alignment variation can be observed.

In total, 52 ANIMAL_CNVR (30 CNL, 21 CNG, and 1 MIX) and 36 POPULATION_CNVR (15 CNL, 7 CNG, and 14 MIX) were identified on 22 and 20 chromosomes (Fig. 5). Visual identification of true positive WGS_CNV found within high confidence CNVR was possible (e.g. Figure 6) as well as the identification of false positive WGS_CNV outside of any high confidence CNVR boundaries (e.g. Figure 7). The total genome covered was 0.22% and 0.24% for ANIMAL_CNVR and the POPULATION_CNVR, respectively. The number of animals carrying a CNVR ranged from one to 25 for the ANIMAL_CNVR and from four to 67 for the POPULATION_CNVR. On one hand, 27 ANIMAL_CNVR (52%) were detected only in one sample. On the other hand, five POPULATION_CNVR (14%) were found in all samples [Suppl. Table 3].

A total of 31 CNVR were found in common between both ANIMAL_CNVR and POPULATION_CNVR sets. The two sets of ANIMAL_CNVR and POPULATION_CNVR were merged for functional analysis to a set of unique high confidence CNVR in which overlapping CNVR were only considered once. Accordingly, 57 regions were considered unique high confidence CNVR. Half of the 57 unique high confidence CNVR were not found in the previous studies of which CNVR were retrieved. These 28 candidate CNVR were all found in the ANIMAL_CNVR set but only 12 of them were part of the POPULATION_CNVR set. No unique high confidence CNVR was found in any gap of the reference assembly.

### Putative functions of the identified CNVR

The 57 unique high confidence CNVR returned 188 Ensembl sequences. Peptide sequences of 160 of those could be retrieved and analyzed with the OmicsBox annotation routine. After analyses, 104 sequences were found that had BLAST hits and could be mapped and annotated for 35 high confidence CNVR. These 35 regions represented 58% of the ANIMAL_CNVR and 61% of the POPULATION_CNVR. Significant GO terms were identified in the three GO main categories biological processes, molecular functions, and cellular component. At the most informative level of the biological processes, 69% of the GO terms related to transcription-related functions and 31% to sensory perception of smell. Of the molecular function terms, 35% related to olfactory receptor activity, 36% to G protein-coupled receptor activity and 29% to binding. Regarding the cellular component terms, 37% of the GO terms were related to membrane components, 36% to cytoplasm elements, and 27% to intracellular elements. Enzyme codes could be retrieved for 10 sequences and connected with 8 KEGG biological pathways (in decreasing number of linked sequences): glycerolipid metabolism, fatty acid elongation, drug metabolism, glycerophospholipid metabolism, nitrogen metabolism, starch and sucrose metabolism, Th1 and Th2 cell differentiation, and T cell receptor signaling pathway. Genes present on the region and their GeneCards information as well as significantly associated GO terms and KEGG pathway are reported for each unique high confidence CNVR in the Suppl. Table 3.

Olfactory receptor genes and immunity-related genes are known to be more often duplicated than other genes and are therefore candidates for CNV studies^70^. Genes of the olfactory receptor family (e.g. *OR5H8*, *OR7A10*, *OLF4*, *OR2AJ*) were contained in six high confidence CNVR on BTA1 (42506338-42587000), BTA7 (8633381–9847400; 10230437–1049220; 41582849–41756370), BTA10 (27020800–27056000), and BTA15 (79748837–79817000). Immunity-related genes were found in five high confidence CNVR: Peptidylprolyl Isomerase A (*PPIA*) was found in a CNVR on BTA1 (93367289–93763299), T Cell Receptor Delta Locus (*TRD*) was found in a CNVR on BTA10 (22340671–22570397), Complement Factor H (*CFH*) and referentially Expressed Antigen In Melanoma (*PRAME*) were found in a CNVR on BTA16 (5701395–5889597 and 54476201–54495676, respectively), and five genes of the *DEFB* family (*DEFB103A*, *DEFB*, *DEFB1*, *DEFB402*, *DEFB4A*) were found in a CNVR on BTA27 (6696801–7186762). Moreover, a CNVR on BTA7 (69606401–69655411) was found to be associated with two KEGG pathways related to immunity function: Th1 and Th2 cell differentiation and T cell receptor signaling. Finally, three regions were found that contained genes related to embryonic development (*RET*, *COL27A1*, *POPDC3*); BTA8 (103487143–103513600), BTA9 (44726004–44875567), BTA28 (13354073–13504624), and eight genes of the homeobox A family contained in one region on BTA4 (68808125–68911600). Genes of the homeobox A family are known for their important role in embryonic development in all mammalian species^71^ [Suppl. Table 3].

## Discussion

In a first step of this study, the whole-genome sequences of 96 Holstein animals were aligned and the genotype array variant positions of 109 Holstein animals (the same 96 plus 13 bulls for which only HD genotype information was available) were updated to the bovine reference genome ARS-UCD1.2. In a second step, copy number variants were identified on the WGS and the GEN data of all animals on a per sample basis. High confidence CNVR were then created *in silico* following two approaches and putative function of the resulting CNVR were retrieved. Finally, identification of CNV on the same sample with reduced marker panels was analyzed to estimate false positive CNV discovery rates when those were identified on lower density genotype array information.

No difference between CNV identified from samples with lower or higher read depth could be observed, a verification that the approach used to identify CNV from WGS data used in CNVnator correctly handled the difference in read depth. Applying a read depth based approach to WGS samples with differing coverage has been found to perform better than approaches based on split read or paired end^27^. Reduction in the number of markers included for GEN_CNV identification on a per animal basis was mostly due to the loss of markers when updating their position to ARS-UCD1.2. A few more markers (3.8% to 0.4%, depending on the marker panel), however, were removed because of their low GC scores. The remaining markers were still distributed evenly on the genome, which allowed for further analyses of the data.

Although relying on the same samples, CNV identification on GEN and WGS data resulted in different sets of variants: more than 250 times more CNV were identified from the WGS data when compared with the GEN_CNV and the total length of the latter was six times higher than that of the WGS_CNV. Such differences in CNV number and length between CNV identified on WGS and GEN information were already described in a study by Zhan *et al*.^24^. Both measures were different due to the large difference in resolution between GEN and WGS data. GEN discovery only relies on a limited number of genome positions whereas the WGS discovery relies on all 2,716,000 Kb of the sequences^40^. The difference in the number and length of CNV was also due to the fact that breakpoints can only be placed at the position of a marker with GEN data, whereas any position of the genome can be defined as a CNV breakpoint using WGS. Furthermore, difference in the set of WGS_CNV and GEN_CNV comes from the relative versus absolute character of both identification methods. Whereas the CNV identification on GEN first rely only on one nucleotide and its signal intensity at a time, relative change in RD between segments of the genome are considered to identify WGS_CNV. More CNL than CNG were observed in both GEN and WGS CNV sets. Generally, CNL are more often found than CNG irrespective of the type of data used for CNV identification^27,29,35,36^. Although detection of both CNL and CNG is of importance, CNL are predicted to have more influence as they affect gene dosage and can lead to exposure of a normally unexpressed (deleterious) recessive allele^70^. CNL are therefore expected to be found less numerous than CNG due to natural selection against deleterious variants in any species, but still found in higher number with the current CNV identification methods^42,55,72,73^, it can be concluded that CNL are easier to detect than CNG.

Differences in marker densities were not directly accounted for in this study even though four marker panels were used in our analyses (Table 3-3). The number of CNV identified decreased with the number of markers on which the identification relied. In contrast, the CNV length increased when fewer markers were included, as the distance between markers increased. This is in line with the difference in CNV length observed between the GEN_CNV and the WGS_CNV described earlier in this paper. In this analysis, CNV that were identified with a lower density but not with the HD marker panel were considered false positive. False positive discovery rates of 12% for the 50 K and of 22% for the 150 K dataset were estimated. Both estimated false positive discovery rates were higher than those reported previously from studies based on WGS data, which ranged between 2% and 8%^57,74^. The lower resolution and lower precision of the CNV identification on GEN data could explain the higher rates observed in our study. In our study, the false positive identification rates were estimated assuming that any CNV identified with the HD marker panel was a true CNV. This strong assumption probably led to underestimation of the false positive identification rates but as these were already consequent, it is certain that CNV identification using a single method *in silico* on a single set of samples is not robust.

The compromise between high true positive discovery rates and low false discovery rates of CNV against high numbers of non-identified CNV and high number of falsely detected CNV is addressed with various measures or quality control protocols in each newly described *in silico* CNV identification method. However, only experimental verification of the identified CNV with qPCR or FISH analyses, for instance, can be considered true validation^57^. These methods have the disadvantage of being lower throughput, resulting in greater time and cost than *in silico* methods. In this study, sets of CNV were defined to be partly validated (or else said to be of high confidence) when the same region was detected as a CNV in GEN and in WGS data. This methodological consensus approach follows not only the conclusion of Baes *et al*.^75^, who found the highest quality single nucleotide variants when those were identified by multiple software but also the study by Zhan *et al*.^24^ on CNV identification. Two sets of high confidence CNVR were created with the same samples. The main difference between those two sets lies in the level at which the consensus between the GEN and the WGS data is implemented. The high confidence of the ANIMAL_CNVR is gained through overlapping of CNV of one animal that were identified from GEN and WGS. In contrary, the high confidence of the POPULATION_CNVR is gained through overlapping of CNVR from all animals that were identified on the GEN or the WGS. In our study, the sample size is limited (n = 67 after quality control) so that although the term “population” is used in POPULATION_CNVR, the data do not represent the whole Holstein cattle population. However, an experiment following the same method with a greater number of samples could be considered representative of a population and the term is therefore kept throughout this paper. An important parameter used for the identification of high confidence CNVR is the minimum percentage of reciprocal overlap set to consider two variants equals. Depending on the study, requirements vary from any overlap (minimum 1 bp) to overlap values between 50% and 90%^36,54,76^. In order to define this threshold in our study, the number of high confidence CNVR remaining with minimum reciprocal overlap ranging between 10% and 90% was calculated. Apart from a steady reduction of the number of high confidence CNVR with increasing minimum percentages from 20% to 80%, no plateau was observed so that any value in this interval could be selected (Fig. 4). The greater change in number of high confidence CNVR identified with 10% and 90% of minimum reciprocal overlap are probably due to the difference in the range of the length between WGS_CNVR and GEN_CNVR as for example short WGS_CNVR cannot cover the required proportion of the relatively long GEN_CNVR even if they have breakpoints within the GEN_CNVR. Considering the relatively small sample size and the aim to compare two high confidence CNVR sets created with the datasets of the present study, a minimum percentage of reciprocal overlap of 50% was selected for both approaches.

With the variants representing 0.22% and 0.24% of the total genome and the similarity of their length distributions, both sets can first be considered similar. Genome coverage of the CNV reported in previous studies on bovine CNV ranged between 0.1% and 6.0% and led to the conclusion that higher number of samples and higher data resolution (WGS versus GEN) led to higher number of CNV discovered and thus to higher experiment-wise genome coverages^24,34,74,76^. In this study, high confidence CNVR had not only to be identified with both WGS and GEN information but also only relied on 67 samples. The stringent CNVR discovery parameters as well as the relatively small number of samples explain the low coverage values. Differences between the POPULATION_CNVR and the ANIMAL_CNVR, however, were observed at multiple levels. Of the CNVR identified in this study but not previously described, only half were included in the POPULATION_CNVR set, whereas all were included in the ANIMAL_CNVR set. The lower concordance of the CNVR found in the latter can be an indication that this approach is less reliable than the POPULATION_CNVR although low overlap between studies is an expected result^12,26,34–37^. Over 50% of the ANIMAL_CNVR were only found in one sample but some POPULATION_CNVR identified were shared by all samples so that this approach can be considered more inclusive. The weight given to a single sample/data combination with this approach is lower than that in the ANIMAL_CNVR approach, as CNV can be found relying on one data type in one animal and on the other data type in another animal, but still be part of the high confidence POPULATION_CNVR set. Considering that all samples came from the Holstein population, the regions found in all samples could be population-specific. For instance, a region on BTA7 (pos. 41582849–41756370) could be found in a review on selective sweeps linked with animals of the Holstein breeds only^77^.

Annotation and pathway analysis of the unique high confidence CNVR showed results in accordance with previous studies on cattle CNV. Bickhart and Liu^70^ previously described known association in cattle between CNV and traits of importance to the industry related to the immunity of the animals. The olfactory receptor genes family was also described in the same review as a group of candidate genes for higher duplication rates. It is thus no surprise that more than 20% of the unique high confidence CNVR contained genes associated with GO terms or KEGG pathways linked with those two functions. A previous study on Holstein dairy cattle showed SNP regions associated with immunity and reproduction that also contained olfactory receptor genes^78^. In addition to olfactory senses and immunity, CNV have been described that are related directly or indirectly to reproduction traits^14^. Four regions were annotated through GeneCards information with reproductive traits. Remarkably, one of these regions (BTA4;68808125–68911600) contains eight genes of the HoxA gene family. The role of this gene family on embryonic development has been described in a study on Chinese indigenous sheep^79^.

## Conclusions

Although each approach has strengths and flaws, CNV can be identified with high confidence when multiple methods or data sources form the base of the identification. The update of the bovine reference genome to the ARS-UCD1.2 assembly still leads to comparable CNV identification results with the previous assembly (UMD3.1). Population-wide identification allowed identification of variants that were more often already known, were more inclusive as less weight was given to the individual sample CNV, allowed identification of regions common to all samples, and could more often be linked to a putative function than sample-based identified CNV. When relying only on genotyping signal intensity information, CNV identification is possible and the variants can be used in downstream analysis, but high false positive identification rates should not be ignored. Further research on larger datasets is now needed. These studies should target high confidence CNVR with a population-wide approach and account for false positive variants.

## Supplementary information

Legend of the supplementary tables.

Supplementary Table 1.

Supplementary Table 2.

Supplementary Table 3.

## Data Availability

Raw sequence data of the CCGP, EDGP and CHE have been deposited to public databases (Sequence Read Archive (SRA) accession SRP017441 for CCGP and accession SRP153409 for EDGP, and European Nucleotide Archive (ENA) project ID PRJEB18113, respectively). For access to the genotypes, interested researchers are asked to contact the owner of the data directly.
