# Supplementary material for: High confidence copy number variants identified in Holstein dairy cattle from whole genome sequence and genotype array data
Source: Sci Rep. 2020 May 15;10:8044. doi: 10.1038/s41598-020-64680-3 (PMC7229195; doi:10.1038/s41598-020-64680-3)
Supplement: Supplementary file 1 — Legend of the supplementary tables. [file 41598_2020_64680_MOESM1_ESM.pdf]

High confidence copy number variants identified in Holstein dairy cattle from whole genome sequence and genotype array data

Adrien M. Butty[1], Tatiane C. S. Chud[1], Filippo Miglior[1], Flavio S. Schenkel[1], Arun Kommadath[2,3], Kirill Krivushin[2] Jason R. Grant[2], Irene M. Häfliger[4], Cord Drögemüller[4], Angela Cánovas[1], Paul Stothard[2], and Christine F. Baes[1,4*]

[1]Centre for Genetic Improvement of Livestock, University of Guelph, Guelph, ON, Canada

[2]Dept of Agricultural, Food and Nutritional Science, University of Alberta,

Edmonton, AB, Canada

[3]Lacombe Research and Development Centre, Agriculture and Agri-Food Canada, Lacombe, AB, Canada

[4]Institute of Genetics, Vetsuisse Faculty, University of Bern, Bern, BE, Switzerland

*Corresponding author: Prof. Dr. Christine Baes
cbaes@uoguelph.ca

Supplementary information – Legends:

Supplementary Table 1: SRA Accession numbers, number of reads mapped, duplicate rate and read depth of the WGS samples

Supplementary Table 2: Genotype array, number of markers for CNV identification, and number of CNV identified of the GEN samples

Supplementary Table 3: Positions, type, number of supporting samples, number of ENSEMBL genes, gene names and putative link to cattle traits of the unique CNVR
